# Association of education level with the risk of female breast cancer: a prospective cohort study

**DOI:** 10.1186/s12905-023-02245-y

**Published:** 2023-03-07

**Authors:** Runxue Jiang, Xia Wang, Zhiguo Sun, Shouling Wu, Shuohua Chen, Haifeng Cai

**Affiliations:** 1 Department of Oncology Surgery, Tangshan People’s Hospital, Tangshan, 063000 China; 2 Department of Gynaecology, Tangshan Hongci Hospital, Tangshan, 063000 China; 3 Health Department of Kailuan(Group), Tangshan, 063000 China

**Keywords:** Education level, Breast cancer, Cohort study

## Abstract

**Background:**

Breast cancer is a serious threat to female health, and its incidence varies with education level (EL). In the present study, the association between EL and the risk of developing female breast cancer was investigated.

**Methods:**

From May 2006 to December 2007, 20,400 observation subjects in Kailuan Cohort received questionnaires and were subjected to clinical examinations for data collection on baseline population characteristics, height, weight, lifestyle and past disease history. Then, these participants were followed up with from the date of recruitment to December 31, 2019. Cox proportional risk regression models were used to analyse the association between EL and the risk of developing female breast cancer.

**Results:**

The cumulative follow-up period of 20,129 observation subjects that meet the inclusion criteria of this study was 254,386.72 person-years, and the median follow-up time was 12.96 years. During the follow-up period, 279 cases of breast cancer were diagnosed. In comparison with the low EL group, the risk of developing breast cancer was significantly higher in the medium (hazard ratio [HR] (95% confidence interval [CI]) = 2.23 (1.12–4.64)] and high [HRs (95% CI) = 2.52 (1.12–5.70)] EL group.

**Conclusion:**

An increased risk of breast cancer was associated with a higher EL, and some certain factors, such as alcohol use and hormone therapy, may play a mediating role.

## Introduction

Breast cancer poses a serious threat to female health worldwide. Based on GLOBOCAN data, in 2020 alone, the number of new cases of breast cancer reached 2.26 million, and the incidence of breast cancer ranks first among female malignant tumours [1]. Although breast cancer is highly dangerous, its aetiology remains unclear, and its known risk factors include genetic factors [2], lack of physical activity, unhealthy weight, alcohol intake, and hormone replacement therapy [3].

Notably, the incidence of malignant tumours varies with the education level (EL). A negative correlation has been observed between EL and the risk of developing gastric, liver, lung, and oesophageal cancer in humans [4, 5], the association of EL with breast cancer remains controversial. EL is associated with an increased risk of breast cancer[5, 6], while other studies have yielded contradictory results [7]. The association between EL and breast cancer was determined by analysing the effect of EL on breast cancer in the population from the Kailuan Study.

## Materials and methods

### Study cohort

The Kailuan Study, which began in 2006, is an ongoing observational cohort study of a functional community population. The EL information of the Kailuan Study subjects was obtained at baseline (from May 2006 to December 2007). Then, continuous follow-up data, including breast cancer data, were obtained from the establishment of the cohort to the present, allowing us to study the association between EL and breast cancer. More details about the Kailuan Study can be found in the literature [8, 9]. Subjects who met the following criteria were recruited: (1) participation in the baseline survey of the Kailuan Study; (2) complete data on EL; and (3) signed the informed consent form. Those with history of malignancy were excluded. The study was approved by the Ethics Committee of Kailuan General Hospital and was conducted in accordance with the Declaration of Helsinki.

### Collection of exposure information

Face-to-face questionnaire interviews and health examinations were performed by well-trained physicians or nurses by using a standardized protocol to gather information at baseline. These baseline data included sociodemographic characteristics (e.g., age and EL), lifestyle characteristics (smoking, alcohol consumption, salt intake and physical activity), history of previous diseases (hypertension, diabetes mellitus and malignant tumour) and physical examination data (height and weight). Participants were asked to take off their hats and shoes and wear thin clothes during the measurement. Height and weight were measured, and body mass index (BMI) was calculated as weight (kg)/height^2^ (m^2^).

### Definition of variables

EL was classified into three categories, namely, low EL (illiteracy and primary), medium EL (middle school) and high EL (college and above). Smoking was defined as smoking one or more cigarettes per week in a recent year, and participants were classified into never smokers, former smokers and current smokers. Alcohol drinking was defined as drinking one or more alcoholic drinks per month for no less than six consecutive months, and participants were classified into never drinkers, former drinkers and current drinkers. Physical activity was classified according to the frequency of physical activity (20 min = 1 instance of activity) performed during leisure time. The categories of physical activity include exercise occasionally (< four instances of activity per week) and frequent exercise (≥ four instances of activity per week).

### Collection of endpoint event information

The follow-up period was considered from the establishment of Kailuan Cohort (May 2006 – December 2007) to December 31, 2019. The follow-up endpoint was newly diagnosed breast cancer or death in the observed subjects (whichever came first). First, information on the participant medical records was obtained through the Tangshan City health insurance system. Then, the medical histories of subjects were collected by professionally trained investigators. Pathology, imaging (including magnetic resonance imaging, computed tomography, and colour Doppler ultrasonography) and blood biochemical examination results were verified by clinicians to confirm the diagnosis of breast cancer. Tumour cases were coded according to the International Classification of Diseases-10 (ICD-10), and breast cancer was coded as C50. Information on fatal events was obtained from the Kailuan Group social insurance system.

### Statistical methods

SAS 9.4 was used to perform statistical analysis of the data. Continuous variables were expressed as mean ± standard deviation, while categorical variables were expressed as the number of cases (percentage). Differences in baseline demographic characteristics among the low, medium and high EL groups were analysed using variance analysis and Chi square test. A multivariable Cox proportional hazards regression model was applied to analyse the associations between EL and the risk of developing breast cancer. Subgroup analysis was performed according to age, BMI, physical activity and hypertension history, and the associations between EL and the risk breast cancer were explored in different subgroups. Sensitivity analysis was conducted to evaluate the consistency of our findings after excluding participants new breast cancer within 2 years of the start of the follow-up period. All statistical tests were considered statistically significant at *P* < 0.05 (two-sided).

## Results

### Baseline characteristics

The number of female participants in the 2006 baseline survey was 20,400. A total of 116 individuals with a history of malignancy and 155 participants lacking baseline EL data were excluded, and 20,129 female participants were eventually included in the statistical analysis. The mean age of the observed subjects was 48.75 ± 11.50 years. The low, medium and high EL groups included 1,078, 16,833 and 2,218 subjects, respectively. In comparison to the medium- and high-EL groups, the mean age, BMI and the proportions of subjects with current smoking, heavy salt intake, frequent physical activity, hypertension and diabetes were higher in the low EL group, whereas the proportions of current drinkers were lower (all *P* values < 0.0001; Table 1).

**Table 1:** Baseline characteristics of the participants

Characteristics	Total cohort (*n* = 20,129)	Low EL (*n* = 1,078)	Medium EL (*n* = 16,833)	High EL (*n* = 2,218)	*P*
Age(years, mean ± SD)	48.75 + 11.50	58.51 + 8.26	49.38 + 10.72	39.29 + 12.51	0.000
BMI(kg/m^2^, mean ± SD)	24.66 + 3.79	26.33 + 3.88	24.78 + 3.75	22.95 + 3.45	0.000
Smoking status, n(%)					
Never	19,662(97.68)	961(89.15)	16,502(98.03)	2199(99.14)	0.000
Former	89(0.44)	31(2.88)	51(0.30)	7(0.32)	
Current	378(1.88)	86(7.98)	280(1.66)	12(0.54)	
Alcohol consumption, *n*(%)					
Never	18,744(93.12)	1018(94.43)	15,919(94.57)	1807(81.47)	0.000
Former	84(0.42)	5(0.46)	48(0.29)	31(1.40)	
Current	1301(6.46)	55(5.10)	866(5.14)	380(17.13)	
Salt intake, *n*(%)					
Light	1888(9.38)	164(15.21)	1408(8.36)	316(14.25)	0.000
General	16,882(83.87)	791(73.38)	14,406(85.58)	1685(75.97)	
Heavy	1359(6.75)	123(11.41)	1019(6.05)	217(9.78)	
Physical activities, *n*(%)					
Occasionally	17,445(86.67)	777(72.08)	14,697(87.31)	1971(88.86)	0.000
Frequently	2684(13.33)	301(27.92)	2136(12.69)	247(11.14)	
Hypertension, *n*(%)					
No	13,563(67.38)	466(43.23)	11,143(66.20)	1954(88.10)	0.000
Yes	6566(32.62)	612(56.77)	5690(33.80)	264(11.90)	
Diabetes mellitus, *n* (%)					
No	18,503(91.92)	899(83.40)	15,445(91.75)	2159(97.34)	0.000
Yes	1626(8.08)	179(16.60)	1388(8.25)	59(2.66)	

*EL* Education level

*P <0.001*

### Association between EL and risk of breast cancer

The cumulative follow-up period in this study was 254,386.72 person-years, with a median follow-up time of 12.96 years. A cumulative total of 279 new breast cancer (*n* = 8 in the low EL group; *n* = 244 in the medium EL group; *n* = 27 in the high EL group) were detected during the follow-up period. Cox proportional risk regression analysis was carried out with the occurrence of breast cancer as the dependent variable and EL difference as the independent variable. In comparison with the low EL group, model 1 was adjusted for age, while model 2 was adjusted for smoking, alcohol consumption, BMI, physical activity, salt intake, hypertension and diabetes mellitus based on model 1. The hazard ratios (HRs) and 95% confidence intervals (95% CI) for breast cancer in the medium and high EL group were 2.23(1.12–4.64) and 2.52(1.12–5.70), respectively. In the sensitivity analysis, after excluding participants who developed breast cancer within 2 years after the start of the follow-up period, the risk of breast cancer was significantly higher in the medium and high EL group (Table 2).

**Table 2:** Associations between EL and breast cancer risk

EL	Total cases	Person years	Incident cases	HR(95%CI)	
				Model1	Model 2
Low	1078	13,467.46	8	Ref	Ref
Medium	16,833	213,025.99	244	2.37(1.17–4.81)	2.23(1.12–4.64)
High	2218	27,893.27	27	2.51(1.12–5.64)	2.52(1.12–5.70)
Excluding participants who developed breast cancer within the first 2 years of follow-up					
Low	1075	13,466.04	5	Ref	Ref
Medium	16,805	213,001.34	216	3.23(1.33–7.87)	3.13(1.28–7.67)
High	2214	27,889.00	23	3.19(1.19–8.54)	3.21(1.19–8.67)

*EL* Education level

Compared with the low El group respectively

Model1 was adjusted for age; model2 was further adjusted for smoking status, alcohol consumption, BMI, physical activity, salt intake, hypertension, and diabetes mellitus

### Subgroup analysis of EL and risk of breast cancer

Subgroup analysis was performed according to age, BMI, physical activity and hypertension history, and the associations between EL and the risk breast cancer was explored in different subgroups. In comparison with the low EL group, in participants aged 50 years and above and the exercise occasionally subgroups, an increased risk of breast cancer was observed in the patients in the medium and high EL group, while an increased risk of breast cancer was observed in high EL’s subjects with BMI ≥ 28 and hypertension subgroup (Table 3).

**Table 3:** Subgroup analysis of the association between EL and breast cancer risk

Variable	EL	B	SE	WALD	*P*	HR(95%CI)
Age (years)						
< 50	medium	0.023	0.716	0.001	0.974	1.02(0.25–4.17)
	high	0.357	0.762	0.219	0.640	1.43(0.32–6.37)
≥ 50	medium	0.968	0.421	5.288	0.022	2.63(1.15–6.01)
	high	1.344	0.534	6.348	0.012	3.84(1.35–10.92)
BMI(kg/m^2^)						
< 28	medium	0.736	0.421	3.057	0.080	2.08(0.92–4.76)
	high	0.716	0.478	2.248	0.134	2.05(0.80–5.22)
≥ 28	medium	1.037	0.725	2.045	0.153	2.82(0.68–11.69)
	high	1.808	0.848	4.549	0.033	6.10(1.16–32.11)
Physical activities						
Occasionally	medium	0.850	0.418	4.133	0.042	2.34(1.03–5.31)
	high	0.995	0.470	4.474	0.034	2.70(1.08–6.80)
Frequently	medium	0.800	0.740	1.169	0.280	2.23(0.52–9.49)
	high	0.571	1.019	0.314	0.575	1.77(0.24–13.04)
Hypertension						
No	medium	0.841	0.587	2.052	0.152	2.32(0.73–7.32)
	high	0.807	0.637	1.605	0.205	2.24(0.64–7.82)
Yes	medium	0.779	0.465	2.806	0.094	2.18(0.88–5.43)
	high	1.385	0.577	5.758	0.016	3.99(1.29–12.39)

*EL* Education level

Compared with the low El group respectively

Adjusted for age, smoking status, alcohol consumption, BMI, physical activity, salt intake, hypertension, and diabetes mellitus

## Discussion

This prospective study based on the Kailuan cohort explored the association between EL and breast cancer risk. The results show that the risk of breast cancer is significantly increased in the medium and high EL groups compared with the low EL group. This study provides reference for the prevention and screening of breast cancer.

The risk of breast cancer significantly increased among subjects with medium and high EL, and this result is consistent with previous research results [5]. In a meta-analysis [6] that includes 18 cohorts, compared with low EL participants, the breast cancer risk of subjects with high EL significantly increased [pooled RR(95%CI) = 1.22 (1.14–1.30)]. In another study, higher EL was associated with a lower risk of breast cancer after adjusting for several confounding factors [7]. These differences in findings may be related to differences in subject race and the length of the follow-up period.

Subgroup analysis results showed that the risk of developing breast cancer is increased in medium and high EL subjects aged ≥ 50 years and exercise occasionally and in high EL subjects with BMI ≥ 28 and hypertension. In a meta-analysis, although the effect was different for pre- and post-menopausal women and for women of different geographic and ethnic groups, a statistically significant association was still observed between hypertension and increased breast cancer risk [10]. Therefore, prevention and screening should be prioritised in the older women. Enhancing physical exercise, controlling BMI level and keeping blood pressure normal are of essential for the prevention and control of breast cancer.

Although the association between EL and breast cancer risk has not been fully understood, several following aspects may tentatively explain the relationship between EL and breast cancer development. Firstly, women with more education are likely to have advanced age at first pregnancy, birth and fewer children, thus increasing their risk of developing breast cancer [11, 12]. Secondly, women with a higher EL are associated with a later menopause onset [13], which is also a breast cancer risk factor [14]. Moreover, more education was correlated with higher alcohol intake and higher prevalence of hormone therapy, and both factors may contribute to the development of breast cancer [6]. In addition, people with higher EL may have better screening compliance, and increased screening can reduce breast cancer mortality but increase incidence [15, 16].

This study has some limitations, such as the absence of some confounders (e.g., use of hormone therapy and age at menopause). The larger study cohort and longer follow-up time aims to minimise the effect of the above missing factors, and the abovementioned variables will be increased in subsequent studies. Secondly, only baseline data of EL were used for the analysis of the association between EL and breast cancer risk, and time-dependent exposures during follow-up were not considered. Some subjects may have received on-the-job education, so changes in EL may affect the risk of developing breast cancer, which should be explored in depth in subsequent studies.

## Conclusion

An increased risk of breast cancer was associated with a higher EL. This condition was more prominent in the sensitivity analysis, and certain factors such as alcohol use and hormone therapy may play a mediating role. Breast cancer can be reduced by reducing alcohol intake, maintaining a normal BMI, controlling blood pressure, increasing physical activity and improving screening compliance.

## Data Availability

The data that support the fndings of this study are available from the Kailuan Study but restrictions apply to the availability of these data, which were used under license for the current study, and so are not publicly available. Data are however available from the authors upon reasonable request and with permission of the Kailuan Study.
